# Association between Pemafibrate Therapy and Triglyceride to HDL-Cholesterol Ratio

**DOI:** 10.3390/jcm11102820

**Published:** 2022-05-17

**Authors:** Teruhiko Imamura, Nikhil Narang, Koichiro Kinugawa

**Affiliations:** 1 The Second Department of Internal Medicine, University of Toyama, Toyama 930-0194, Japan; kinugawa-tky@umin.ac.jp; 2 Advocate Christ Medical Center, Oak Lawn, IL 60453, USA; nikhil.narang@gmail.com

**Keywords:** dyslipidemia, cardiovascular disease, heart failure

## Abstract

Background: Pemafibrate is a novel selective peroxisome proliferator-activated receptor-α modulator, which was demonstrated to reduce serum triglyceride level with few drug-related adverse events in phase II and III clinical trials. However, its clinical implication in real-world practice remains unknown. Triglyceride/HDL-cholesterol ratio is a surrogate of small dense LDL-cholesterol, which is a newly proposed cardiovascular risk factor independent of LDL-cholesterol levels. Methods: Consecutive patients who received pemafibrate between April 2020 and September 2021 and continued therapy for at least 3 months were included in this retrospective analysis. The primary outcome was the trend in triglyceride/HDL-cholesterol ratio during the 3-month treatment period. The change in cardiovascular event rate between the one-year pre-treatment period and the on-treatment period was also analyzed. Results: A total of 19 patients (median age 63 years, 74% men) were included and continued pemafibrate therapy for 3 months without any drug-related adverse events. Sixteen were add-on and three were conversions from other fibrates. Triglyceride/HDL-cholesterol ratio decreased significantly from 5.85 (4.19, 16.1) to 3.14 (2.39, 4.62) (*p* < 0.001). The cardiovascular event rate decreased significantly from 0.632 events/year to 0.080 events/year (*p* < 0.001). Conclusions: Pemafibrate therapy might have the potential to lower triglyceride/HDL-cholesterol ratio and decrease cardiovascular events.

## 1. Background

Low-density lipoprotein (LDL)-cholesterol-lowering therapy with statins can reduce the risk of incident cardiovascular diseases considerably [1], whereas residual risk remains specifically in patients with significant hypertriglyceridemia [2]. Fibrates, which activate peroxisome proliferator-activated receptor alpha (PPARα), are commonly used to decrease triglyceride levels [3], though they carry a risk of adverse side effects by way of drug interactions. They can affect hepatic drug-metabolizing enzyme activity and alter metabolism of statins [4]. Furthermore, triglyceride-lowering therapy with fibrates has not been shown to offer considerable cardiovascular disease risk reduction in large randomized control [5]. 

Pemafibrate is a novel selective PPARα activator that was recently introduced with an expected improvement in efficacy and safety compared with traditional fenofibrate in prior cohort studies [6,7,8,9,10,11,12]. However, the clinical benefit of pemafibrate in real-world practice has not yet been clarified well. In this study, we investigated the impact of pemafibrate therapy on triglyceride/high-density lipoprotein (HDL)-cholesterol ratio, indicating the existence of small dense LDL-cholesterol which is a recently introduced surrogate of cardiovascular risk [13], as well as on changes in cardiovascular event rates following treatment in those with hypertriglyceridemia. 

## 2. Methods

### 2.1. Patient Selection

Consecutive patients who were initiated on pemafibrate for the first time between April 2020 and September 2021 and continued pemafibrate for at least 3 months to treat hypertriglyceridemia were retrospectively included in this study. Some of them received pemafibrate as a first fibrate and others received pemafibrate as conversion from other fibrates. Those who received pemafibrate previously or those who initiated pemafibrate before the study period were excluded. Written informed consents were obtained from all participants before the listing. The institutional review board approved the study protocol (R2015154, 11 April 2016). 

### 2.2. Biomarker Measurement

Laboratory data including lipid parameters were assayed by standard laboratory procedures. All serum and plasma samples were obtained just before the initiation of pemafibrate as baseline data and 3 months later as follow-up data in a fasting condition and frozen at −80 degrees immediately. We defined the change in triglyceride level relative to HDL-cholesterol as the primary outcome. Triglyceride-rich lipoprotein-cholesterol was calculated as follows: total cholesterol−(LDL-cholesterol and HDL-cholesterol) [14].

### 2.3. Other Clinical Data

Demographics, comorbidities, and medication data were obtained just before pemafibrate initiation as baseline characteristics. Cardiovascular events including heart failure, stroke, and ischemic heart disease were counted during the one-year pre-treatment period and during the pemafibrate treatment period. The change in cardiovascular event rate stratified by time of pemafibrate initiation was defined as the secondary outcome. 

### 2.4. Statistical Analysis

Continuous variables are presented as median (lower quartile, higher quartile) irrespective of their distribution. Categorical variables are presented as numbers and percentages. The trend in continuous variables was assessed by Wilcoxon signed-rank test. Event rates were compared between the pre-treatment period and the treatment period by negative binomial regression analysis. A value of *p* < 0.05 was considered statistically significant. Statistical analyses were performed using SPSS Statistics 23 (SPSS Inc., Armonk, IL, USA).

## 3. Results

### 3.1. Baseline Characteristics

A total of 19 patients were included. Median age was 63 (54, 67) years old and 74% were men (Table 1). Thirteen patients had hypertension and twelve had diabetes mellitus. All patients had dyslipidemia and continued pemafibrate therapy for over 3 months (median 290 (100, 365) days) without any drug-related adverse events including rhabdomyolysis, acute kidney injury, hepatic injury, and worsening of diabetes mellitus. The doses of pemafibrate remained unchanged during the observational period. Other anti-dyslipidemia agents were not changed during the observational period. 

### 3.2. Trends in Triglyceride/HDL-Cholesterol Ratio (Primary Endpoint)

**Figure 1:**
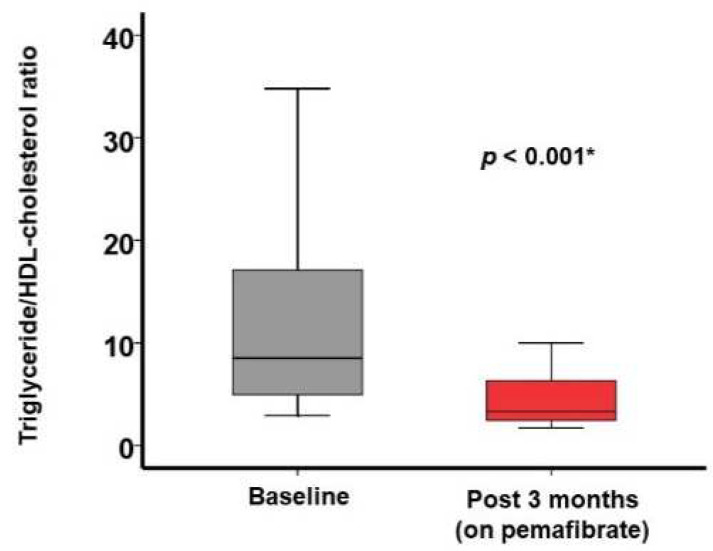
Triglyceride/HDL-cholesterol ratio at baseline and 3 months following the initiation of pemafibrate. * *p* < 0.05 by Wilcoxon signed-rank test.

Following 3-month pemafibrate therapy, triglyceride level decreased from 304 (197, 589) mg/dL to 156 (117, 2154) mg/dL (*p* < 0.001; Table 2). HDL-cholesterol significantly increased from 46 (36, 49) mg/dL to 49 (41, 55) mg/dL (*p* = 0.001). Triglyceride/HDL-cholesterol ratio decreased from 5.85 (4.19, 16.1) to 3.14 (2.39, 4.62) (*p* < 0.001; Figure 1). 

**Figure 2:**
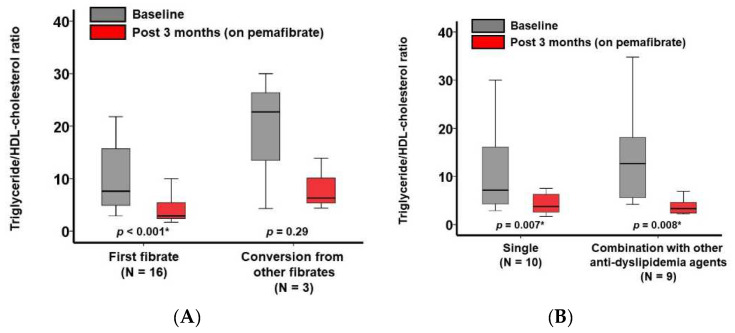
Triglyceride/HDL-cholesterol ratio at baseline and 3 months following the initiation of pemafibrate stratified by the types of pemafibrate use (as a first pemafibrate and conversion from other fibrates) (**A**) and the existence of concomitant lipid-lowering medications (**B**) * *p* < 0.05 by Wilcoxon signed-rank test.

Sixteen patients received pemafibrate as a first pemafibrate and the other three received pemafibrate as a conversion from other fibrates. The triglyceride/HDL-cholesterol ratio decreased significantly irrespective of the conversion from other fibrates (*p* < 0.05 for both; Figure 2A). 

Ten patients did not receive any other anti-dyslipidemia agents except for pemafibrate, and the other nine received other dyslipidemia therapies including statins. There were no statistically significant differences in baseline characteristics between the two groups (*p* > 0.05 for all; Table 1). Triglyceride/HDL-cholesterol ratio decreased significantly irrespective of the concomitant use of other dyslipidemia therapies (*p* < 0.05 for both; Figure 2B).

Twelve patients had diabetes mellitus. The baseline triglyceride/HDL-cholesterol ratio was statistically not different between those with and without diabetes mellitus (*p* = 0.26). Following pemafibrate therapy, the ratio decreased significantly irrespective of the existence of diabetes mellitus (*p* < 0.05 for both).

### 3.3. Trends in Other Laboratory Data

Hemoglobin, estimated glomerular filtration ratio, and plasma B-type natriuretic peptide levels remained unchanged during the 3-month pemafibrate therapy (*p* > 0.05 for all; Table 2). Urine protein level decreased significantly from 0.03 (0.00, 0.48) g/g creatinine to 0.00 (0.00, 0.21) g/g creatinine (*p* = 0.021). 

### 3.4. Trends in Cardiovascular Event Rate

**Figure 3:**
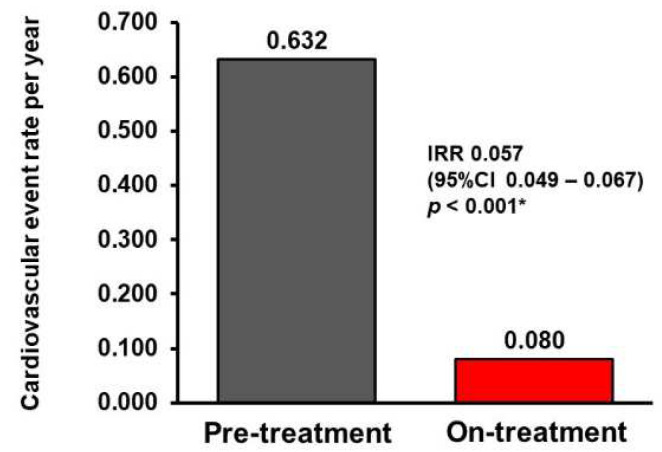
Comparison in the cardiovascular event rates between the pre-treatment period and the on-treatment period. IRR, incidence rate ratio; CI, confidence interval. * *p* < 0.05 by negative binomial regression analyses.

During the 1-year observational period before the administration of pemafibrate, there were 12 cardiovascular events (7 worsening heart failure events, 3 unstable ischemic heart disease events, and 2 acute kidney injury events). During the pemafibrate therapy (median 290 (100, 365) days), one cardiovascular event (worsening heart failure) was observed. Event rates decreased from 0.632 events per year to 0.080 events per year (incidence rate ratio 0.057, 95% confidence interval 0.049–0.067, *p* < 0.001; Figure 3).

## 4. Discussion

In this retrospective study, we investigated the efficacy of pemafibrate therapy in real-world clinical practice in patients with a variety of comorbidities. (1) Three-month pemafibrate therapy decreased triglyceride/HDL-cholesterol ratio significantly. (2) Urine protein level decreased following the 3-month pemafibrate therapy. (3) The cardiovascular event rate was lower during the pemafibrate therapy compared to the pre-treatment period. 

Pemafibrate may be a superior alternative to conventional fibrates, including fenofibrate, in improving hypertriglyceridemia, maintaining relatively lower drug-related adverse event rates [15]. The safety and efficacy of pemafibrate were demonstrated in large-scale studies with carefully selected cohorts (randomized control trials and observation studies) including those with diabetes mellitus and chronic kidney disease [6,7,8,9,10,11,12]. In the phase III trial, pemafibrate 0.1 mg/day, 0.2 mg/day, and 0.4 mg/day were superior to fenofibrate 200 mg/day in adverse events and adverse drug reactions. 

We validated the efficacy and safety of pemafibrate therapy in our real-world clinical practice in patients with a variety of comorbid conditions. All patients who received pemafibrate were included without any strict restrictions. All patients continued pemafibrate therapy without drug-related adverse events that required drug termination. 

We focused on the impact of pemafibrate on triglyceride/HDL-cholesterol ratio, instead of triglyceride alone. The triglyceride/HDL-cholesterol ratio is a recently-introduced surrogate of small dense LDL-cholesterol [13], which is not measured routinely in clinical care. Small dense LDL-cholesterol is vulnerable to oxidative stress and as a result highly atherogenic [16]. Additionally, the presence of small dense LDL-cholesterol is independently associated with an increased risk of adverse cardiovascular events independent of LDL-cholesterol [17,18].

Although we did not measure small dense LDL-cholesterol directly, three-month pemafibrate therapy may decrease the burden of small dense LDL-cholesterol levels, irrespective of the concomitant lipid-lowering therapy or conversion from other fibrates. 

Furthermore, we observed in this study that pemafibrate decreased triglyceride-rich lipoprotein-cholesterol, which was very recently introduced and demonstrated to be associated with incremental cardiovascular risk [14].

We observed a decrease in urine protein levels following three months of pemafibrate therapy. A few studies have investigated the impact of pemafibrate on renal function in the clinical setting. In mice with diabetic nephropathy, pemafibrate therapy decreased albuminuria via inhibition of renal lipid content and oxidative stress [19]. In another experimental study using mice with fatty acid overload associated nephropathy, pemafibrate therapy was associated with reno-protective effects via the activation and maintenance of renal fatty acid metabolism [20]. Further studies including those with nephropathy accompanying considerable proteinuria are warranted to investigate the reno-protective impact of pemafibrate. 

Understanding that the presence of small dense LDL-cholesterol increases cardiovascular risk independent of LDL-cholesterol level, it is reasonable to hypothesize that pemafibrate would reduce cardiovascular events by lowering small dense LDL-cholesterol. We observed a lower cardiovascular event rate during pemafibrate therapy compared to the pre-treatment period (without pemafibrate). An ongoing PROMINENT study [21], which is prospectively investigating the impact of pemafibrate therapy on reducing cardiovascular events compared to placebo in patients with diabetes mellitus and hypertriglyceridemia, will serve to test this hypothesis. Target thresholds of triglyceride, triglyceride/HDL-cholesterol ratio, and small dense LDL-cholesterol, as well as triglyceride-rich lipoprotein-cholesterol levels during pemafibrate therapy, remains an area of future investigation. 

Our study was comprised of a small cohort size with short-term observational period. Given the small sample size, we assumed all variables as non-parametric data irrespective of their distribution. Non-significance in this study does not indicate similarity. This is a proof-of-concept preliminary study, and further larger-scale studies are warranted to validate and expand our findings, although it should take longer clinical experience of pemafibrate therapy in real-world practice. We lack a control group and just compared the clinical variables between baseline and 3 months later. The medications including pemafibrate remained unchanged during the treatment period, but other confounders might have affected the outcomes. Given the retrospective nature of this study and a lack of control group, we observed just a potential association between pemafibrate therapy and clinical variables, but cannot infer causality. 

## 5. Conclusions

Pemafibrate therapy might be associated with a lower triglyceride/HDL-cholesterol ratio and risk of incident cardiovascular events, although definite conclusions cannot be derived from this small sample-sized study. Larger-scale longer-term studies are warranted to validate and strengthen our findings. Of note, further trials are needed to better assess the reduction in the degree of atherosclerosis, lipid parameters including small dense LDL-cholesterol, and cardiovascular risk in patients receiving pemafibrate therapy.

## Figures and Tables

**Table 1:** Baseline characteristics.

	Total (N = 19)	Pemafibrate Alone (N = 10)	Combination with other Agents (N = 9)	*p* Value
Demographics				
Age, years	63 (54, 67)	61 (40, 64)	61 (54, 72)	
Men	14 (74%)	7 (70%)	7 (78%)	0.73
Body mass index, kg/m^2^	23.2 (21.1, 25.7)	23.1 (20.9, 24.5)	23.3 (21.1, 25.3)	0.36
Comorbidity				
Hypertension	13 (68%)	6 (60%)	7 (78%)	0.41
Dyslipidemia	19 (100%)	10 (100%)	9 (100%)	1
Diabetes mellitus	12 (63%)	5 (50%)	7 (78%)	0.21
Atrial fibrillation	9 (47%)	5 (50%)	4 (44%)	0.81
Fibrate use				
As a first fibrate	16 (74%)	7 (70%)	9 (100%)	0.073
As a conversion from other fibrates	3 (16%)	3 (30%)	0 (0%)	0.073

Continuous variables are presented as median (lower quartile, higher quartile). Categorical variables are presented as numbers and percentages.

**Table 2:** Trends in laboratory data.

	Baseline	3 Months	*p* Value
Lipid parameters			
Triglyceride, mg/dL	304 (197, 589)	156 (117, 254)	<0.001 *
LDL-cholesterol, mg/dL	107 (75, 127)	97 (79, 141)	0.39
HDL-cholesterol, mg/dL	46 (36, 49)	49 (41, 55)	0.001 *
Triglyceride-rich lipoprotein-cholesterol, mg/dL	61 (39, 73)	25 (21, 41)	<0.001 *
Total cholesterol, mg/dL	212 (164, 220)	187 (151, 211)	0.016 *
Other laboratory data			
Hemoglobin, g/dL	14.5 (12.6, 16.0)	13.7 (12.7, 15.2)	0.1
Estimated glomerular filtration ratio, mL/min/1.73 m^2^	67.1 (38.9, 74.5)	64.7 (36.1, 81.6)	0.64
Plasma B-type natriuretic peptide, pg/mL	73 (65, 89)	43 (34, 44)	0.11
Serum C-reactive protein, mg/dL	0.26 (0.11, 0.29)	0.21 (0.08, 0.25)	0.076
Urine protein, g/g creatinine	0.03 (0.00, 0.48)	0.00 (0.00, 0.21)	0.021 *

LDL, low-density lipoprotein; HDL, high-density lipoprotein. Continuous variables are presented as median and interquartile and compared between the two groups using Wilcoxon signed-rank test. * *p* <0.05.

## Data Availability

Data including study protocol are available from the corresponding authors upon reasonable request.

## References

[1] Kazi D.S., Penko J.M., Bibbins-Domingo K. (2017). Statins for Primary Prevention of Cardiovascular Disease: Review of Evidence and Recommendations for Clinical Practice. Med. Clin. N. Am..

[2] Fulcher J., O’Connell R., Voysey M., Emberson J., Blackwell L., Mihaylova B., Simes J., Collins R., Kirby A., Cholesterol Treatment Trialists’ (CTT) Collaboration (2015). Efficacy and safety of LDL-lowering therapy among men and women: Meta-analysis of individual data from 174,000 participants in 27 randomised trials. Lancet.

[3] Barter P.J., Rye K.A. (2006). Cardioprotective properties of fibrates: Which fibrate, which patients, what mechanism?. Circulation.

[4] Ferri N., Corsini A., Sirtori C., Ruscica M. (2017). PPAR-alpha agonists are still on the rise: An update on clinical and experimental findings. Expert Opin. Investig. Drugs.

[5] Ginsberg H.N., Elam M.B., Lovato L.C., Crouse III J.R., Leiter L.A., Linz P., Friedewald W.T., Buse J.B., Gerstein H.C., The ACCORD Study Group (2010). Effects of combination lipid therapy in type 2 diabetes mellitus. N. Engl. J. Med..

[6] Araki E., Yamashita S., Arai H., Yokote K., Satoh J., Inoguchi T., Nakamura J., Maegawa H., Yoshioka N., Tanizawa Y. (2018). Effects of Pemafibrate, a Novel Selective PPARalpha Modulator, on Lipid and Glucose Metabolism in Patients With Type 2 Diabetes and Hypertriglyceridemia: A Randomized, Double-Blind, Placebo-Controlled, Phase 3 Trial. Diabetes Care.

[7] Araki E., Yamashita S., Arai H., Yokote K., Satoh J., Inoguchi T., Nakamura J., Maegawa H., Yoshioka N., Tanizawa Y. (2019). Efficacy and safety of pemafibrate in people with type 2 diabetes and elevated triglyceride levels: 52-week data from the PROVIDE study. Diabetes Obes. Metab..

[8] Yokote K., Yamashita S., Arai H., Araki E., Suganami H., Ishibashi S., On Behalf of the K-Study Group (2019). Long-Term Efficacy and Safety of Pemafibrate, a Novel Selective Peroxisome Proliferator-Activated Receptor-alpha Modulator (SPPARMalpha), in Dyslipidemic Patients with Renal Impairment. Int. J. Mol. Sci..

[9] Arai H., Yamashita S., Yokote K., Araki E., Suganami H., Ishibashi S., on behalf of the K-877 Study Group (2018). Efficacy and Safety of Pemafibrate Versus Fenofibrate in Patients with High Triglyceride and Low HDL Cholesterol Levels: A Multicenter, Placebo-Controlled, Double-Blind, Randomized Trial. J. Atheroscler. Thromb..

[10] Arai H., Yamashita S., Yokote K., Araki E., Suganami H., Ishibashi S., K-877 Study Group (2017). Efficacy and safety of K-877, a novel selective peroxisome proliferator-activated receptor alpha modulator (SPPARMalpha), in combination with statin treatment: Two randomised, double-blind, placebo-controlled clinical trials in patients with dyslipidaemia. Atherosclerosis.

[11] Ishibashi S., Yamashita S., Arai H., Araki E., Yokote K., Suganami H., Fruchart J.-S., Kodama T., K-877-04 Study Group (2016). Effects of K-877, a novel selective PPARalpha modulator (SPPARMalpha), in dyslipidaemic patients: A randomized, double blind, active- and placebo-controlled, phase 2 trial. Atherosclerosis.

[12] Ishibashi S., Arai H., Yokote K., Araki E., Suganami H., Yamashita S., K-877 Study Group (2018). Efficacy and safety of pemafibrate (K-877), a selective peroxisome proliferator-activated receptor alpha modulator, in patients with dyslipidemia: Results from a 24-week, randomized, double blind, active-controlled, phase 3 trial. J. Clin. Lipidol..

[13] Maruyama C., Imamura K., Teramoto T. (2003). Assessment of LDL particle size by triglyceride/HDL-cholesterol ratio in non-diabetic, healthy subjects without prominent hyperlipidemia. J. Atheroscler. Thromb..

[14] Vallejo-Vaz A.J., Fayyad R., Boekholdt M., Hovingh G.K., Kastelein J.J., Melamed S., Barter P., Waters D.D., Ray K.K. (2018). Triglyceride-Rich Lipoprotein Cholesterol and Risk of Cardiovascular Events Among Patients Receiving Statin Therapy in the TNT Trial. Circulation.

[15] Yamashita S., Masuda D., Matsuzawa Y. (2020). Pemafibrate, a New Selective PPARalpha Modulator: Drug Concept and Its Clinical Applications for Dyslipidemia and Metabolic Diseases. Curr. Atheroscler. Rep..

[16] Lamarche B., Lemieux I., Despres J.P. (1999). The small, dense LDL phenotype and the risk of coronary heart disease: Epidemiology, patho-physiology and therapeutic aspects. Diabetes Metab..

[17] Arai H., Kokubo Y., Watanabe M., Sawamura T., Ito Y., Minagawa A., Okamura T., Miyamato Y. (2013). Small dense low-density lipoproteins cholesterol can predict incident cardiovascular disease in an urban Japanese cohort: The Suita study. J. Atheroscler. Thromb..

[18] Hoogeveen R.C., Gaubatz J.W., Sun W., Dodge R.C., Crosby J.R., Jiang J., Couper D., Virani S.S., Kathiresan S., Boerwinkle E. (2014). Small dense low-density lipoprotein-cholesterol concentrations predict risk for coronary heart disease: The Atherosclerosis Risk in Communities (ARIC) study. Arterioscler. Thromb. Vasc. Biol..

[19] Maki T., Maeda Y., Sonoda N., Makimura H., Kimura S., Maeno S., Takayanagi R., Inoguchi T. (2017). Renoprotective effect of a novel selective PPARalpha modulator K-877 in db/db mice: A role of diacylglycerol-protein kinase C-NAD(P)H oxidase pathway. Metabolism.

[20] Aomura D., Harada M., Yamada Y., Nakajima T., Hashimoto K., Tanaka N., Kamijo Y. (2021). Pemafibrate Protects against Fatty Acid-Induced Nephropathy by Maintaining Renal Fatty Acid Metabolism. Metabolites.

[21] Pradhan A.D., Paynter N.P., Everett B.M., Glynn R.J., Amarenco P., Elam M., Ginsberg H., Hiatt W.R., Ishibashi S., Koenig W. (2018). Rationale and design of the Pemafibrate to Reduce Cardiovascular Outcomes by Reducing Triglycerides in Patients with Diabetes (PROMINENT) study. Am. Heart J..

